# Monthly Summary

**Published:** 1885-10

**Authors:** 


### Monthly Summary.

Peroxide of Hydrogen.—*Dr. W. W. Allport, Chicago.* —The peroxide of hydrogen ($H_2 O_2$), though not a new remedy, has only within the last few years gained much prominence in the treatment of surgical diseases. One of its uses in dental and oral surgery is in blind or deep-seated abscesses, such as arise from roots of diseased teeth. As the tendency of pus is always downward, when these cases occur in the lower jaw it is not infrequent that the abscess, if left to itself, and sometimes even after the tooth is extracted, will point through the external tissues at the lower margin of the jaw, and occasionally downward between the muscles of the neck, and open at various points, even as low down as the clavicle. The usual treatment is to extract the tooth and evacuate the pus through the alveolus, but it often happens that the formation of pus and the continuance of suppuration is not checked, and the abscess points, or is opened through the external tissue of the face or neck, leaving, when healed, a disfiguring scar.

By injecting peroxide of hydrogen into such abscesses before they point through the external tissues, this serious disfigurement can usually be averted, and the suppurative process is materially shortened. It is also a valuable aid for the evacuation of the purulent contents of the antrum of Highmore, in catarrhal and suppurative inflammations, and especially where the sinuses are divided into two or more pockets by bony septi. These cases are often protracted by the inability of the surgeon to perfectly evacuate them. But with this preparation it becomes a simple matter after access has been gained to the cavity by the extraction of a tooth or the perforation of its external wall in the proper place at the juncture of the cheek with the alveolar border. A free opening must always be made for the escape of the contents, in in order to avoid pressure from the rapid evolution of gas. Two or three applications of a dram each is usually sufficient to completely empty the sac.

It is used with the most gratifying results in the treatment of pyorrhea alveolaris, and is an invaluable agent in treating pulpless teeth, as by its ation all decomposed matter from the pulp chamber and dentinal tubuli is readily ejected, thereby removing the most frequent cause of discoloring of this class of teeth, of inflammation of the peridental membrane, as well as alveolar abscesses.

The efficacy of peroxide of hydrogen depends on the case with which it is decomposed into oxygen and water. Pus is one of the many substances which causes this decomposition. Hydrogen peroxide acts first chemically and then mechanically. When the decomposition takes place the oxygen is set free and escapes from a liquid to a gaseous form; this expansion of the gas distends the pus cavity, and as it escapes from the orifice, it carries much of the pus with it, and its application should be repeated till all purulent accumulations are evacuated. The liberated oxygen, being in a nascent or active condition, rapidly oxidizes the products of suppuration, and destroys many of the micro-organisms of suppuration.* Hence it is a disinfectant and anti-septic.

Finally, peroxide of hydrogen, after its decomposition, leaves no material in the system which is foreign to the system, and it is, therefore, one of the most efficient and harmless disinfectants and anti-septics that can be used, in all forms of purulent inflammation.—*Address at American Medical Association.*

---

ALCOHOLIC PARALYSIS.—The immediate and transient effects of an excessive quantity of alcohol upon the human nervous system, whether they are manifested in the form of drunkenness, or of delirium tremens, or of an acute attack of

*See Gradle on "Bacteria and the Germ Theory of Disease," pp. 39 and 151.

insanity, are well known. Scarcely less evident are the effects produced upon the nervous system by a less excessive, but a more prolonged, abuse of alcoholic drinks. These effects may be manifested either in a general failure of physical and mental power, or in a form of disease closely resembling progressive paralytic dementia, or in various forms of chronic insanity, or in epilepsy, or in neuralgia, or in paralysis. In the acute form of alcoholic poisoning, no change in the structure of the nervous system has been found, except that the meninges in common with the internal organs and the mucous membranes are the seat of a very decided injection and slight exudation. In the chronic form of alcoholism, a number of pathological changes have been discovered in the nervous system, which, however, vary greatly in different cases.

Of late years the paralysis which results from the abuse of alcohol has been accurately described by numerous observers, and the attempt has been made to discover the lesion of the nervous system which is associated with this form of paralysis. Two cases which are reported by Dr. Henry Hun, of Albany, in the *American Journal of Medical Sciences* for April, 1885, are typical examples of this disease, and contribute to a better understanding of it.

Dr. Hun has collected the recorded cases of alcoholic paralysis, and from their study he holds that we are justified in regarding it as a special form of disease with the following symptoms: After a number of cerebral and gastric disturbances due to the alcoholic poisoning, the symptoms of the disease proper commence with neuralgic pains and paræsthesiæ in the legs, which gradually extend to the upper extremities, and which are accompanied at first by hyperæsthesia, later by anæsthesia, and in very severe cases by retardation of conduction of pain. Along with these symptoms appears a muscular weakness, which steadily increases to an extreme degree of paralysis, and is accompanied by rapid atrophy and by great sensitiveness of the muscles to pressure and passive motion. Both the sensory and motor disturbances are symmetrically distributed, and the paralysis attacks especially the extensor muscles. In addition to these motor and sensory symptoms, there is also a decided degree of ataxia. The tendon reflexes are abolished and vaso-motor symptoms, such as œdema, congestion, etc., are usually present. Symptoms of mental disturbance are always present in the form of loss of memory and in transient delirium.

The lesion is in all probability a degeneration of the peripheral nerve fibres and of the nerve cells in the cerebral cortex, together with a chronic congestion or inflammation of the pia mater. This lesion explains well the symptoms, although it is curious that alcohol should not attack the spinal cord, but only the highest and lowest part of the nervous system, if one may so call the cortex of the brain and the terminal branches of the peripheral nerves.—*Detroit Lancet.*

---

NOURISHING THE TISSUES OF THE TEETH.—Dr. Frank Abbott says: For a number of years past I have entertained the views that there was some difficulty existing not due to an insufficiency of lime-salts, which occasions so many faulty and imperfectly formed teeth. With that idea in view I have advised exercise in the open air and other kinds of treatment for some patients which would favorably affect their digestion. That the food ordinarily taken contains sufficient lime-salts to form and to sustain the teeth I have no doubt (except in cases of extreme anemia, during gestation and lactation). I believe the fault is beyond that. There is a lack of proper nourishment of the tissues, due to imperfect digestion, which depends again on the proper "nerve tone." I believe this to be the real cause of the difficulty. It is reasonable to suppose that any *tonic*, whether taken in the form of exercise in the open air, or in any other form, which affects favorably other portions of the body, will affect the teeth favorably as well. When we have *ascertained the functions of the great nerve-centers*, and those functions are assured, then we will probably have more perfectly-formed teeth.—*Items of Interest.*

